# A mitochondrial-associated link between an effector caspase and autophagic flux

**DOI:** 10.4161/auto.32170

**Published:** 2014-08-13

**Authors:** Lindsay DeVorkin, Sharon M Gorski

**Affiliations:** 1 The Genome Sciences Centre; BC Cancer Agency; Vancouver, BC Canada; 2 Department of Molecular Biology and Biochemistry; Simon Fraser University; Burnaby, BC Canada

**Keywords:** caspase, Dcp-1, sesB, adenine nucleotide translocase, mitochondria, autophagy, ATP, mitochondrial dynamics, ovary

## Abstract

It has become evident that caspases function in nonapoptotic cellular processes in addition to the canonical role for caspases in apoptotic cell death. We recently demonstrated that the *Drosophila* effector caspase Dcp-1 localizes to the mitochondria and positively regulates starvation-induced autophagic flux during mid-oogenesis. Loss of Dcp-1 leads to elongation of the mitochondrial network, increased levels of the adenine nucleotide translocase sesB, increased ATP levels, and a reduction in autophagy. We found that sesB is a negative regulator of autophagic flux, and Dcp-1 interacts with sesB in a nonproteolytic manner to regulate its stability, uncovering a novel mechanism of mitochondrial associated, caspase-mediated regulation of autophagy in vivo.

*Drosophila* mid-oogenesis is a powerful model system to study cell death and its crosstalk with autophagy. We previously reported that in addition to its role in cell death during mid-oogenesis, *Drosophila* Dcp-1 (Death caspase-1) is required for starvation-induced autophagy at this stage. However, the requirement for Dcp-1 in starvation-induced autophagic flux, and the mechanism of Dcp-1 mediated autophagy, remained unknown. In our recent study, we examined autophagic flux using ref(2)P, the *Drosophila* homolog of SQSTM1/p62 and marker of autophagic activity, as well as the GFP-mCherry-Atg8a autophagy reporter in mid-stage egg chambers of nutrient-deprived control and *Dcp-1^Prev1^* loss-of-function flies. Whereas degenerating mid-stage egg chambers from control flies show increased autophagic flux, *Dcp-1^Prev1^* flies show a reduction in autolysosomes and increased ref(2)P in degenerating mid-stage egg chambers. Well-fed flies overexpressing Dcp-1 in the germline show increased autophagic flux in both nondegenerating and degenerating mid-stage egg chambers, confirming that Dcp-1 positively regulates autophagic flux during *Drosophila* mid-oogenesis.

Like other effector caspases, Dcp-1 is synthesized as an inactive zymogen, or pro-caspase, that contains an N-terminal domain followed by a large 20-kDa (p20) and a small 10-kDa (p10) subunit separated by a short linker region. Cleavage of the linker domain is required for caspase activation. Unlike most other caspases, Dcp-1 can undergo auto-processing and activate itself. This feature could be key to its nonapoptotic functions where low-level and/or localized caspase activation may be important. Indeed, we found that pro-Dcp-1 localizes within mitochondria, whereas cleaved Dcp-1 localizes to both the mitochondria and cytoplasm. Examination of mitochondrial dynamics in Dcp-1 RNAi treated cells and in ovaries from *Dcp-1^Prev1^* flies revealed that loss of Dcp-1 results in an elongated mitochondrial phenotype even under nutrient-full conditions. This is the first report showing that a *Drosophila* effector caspase regulates mitochondrial dynamics under nonstressed conditions. It was previously reported that during starvation-induced autophagy, mitochondria elongate to sustain ATP levels, suggesting that the elongated mitochondrial phenotype in *Dcp-1^Prev1^* flies may be associated with increased ATP production. We found that ovaries from *Dcp-1^Prev1^* flies contain increased ATP levels under both fed and starvation conditions. Furthermore, inhibition of the mitochondrial ATP synthase using oligomycin A results in increased autophagic activity in degenerating mid-stage egg chambers following starvation. These data demonstrate a novel role for an effector caspase in mediating mitochondrial dynamics and ATP levels in both basal and nutrient stress conditions in vivo.

In light of our observations, we hypothesized that Dcp-1 may regulate a mitochondrial protein involved in ATP synthesis or transport as a mechanism to control ATP levels and autophagy. We observed that sesB, a mitochondrial adenine nucleotide translocase, is decreased following starvation in wild-type flies, whereas sesB is increased in ovaries from *Dcp-1^Prev1^* flies under both fed and starvation conditions. This indicated that sesB may negatively regulate autophagic flux and could be itself negatively regulated by Dcp-1. Analysis of *sesB^Org^* hypomorphic flies confirmed that sesB normally functions to suppress autophagic flux and cell death during mid-oogenesis. Immunoprecipitation assays showed that pro-Dcp-1 interacts with sesB in the mitochondria under basal conditions. We were unable to detect sesB cleavage fragments in Dcp-1 in vitro cleavage assays suggesting Dcp-1 likely associates with sesB in a nonproteolytic manner. It is possible, however, that low levels of active Dcp-1 interact with sesB but are undetectable, or alternatively, perhaps pro-Dcp-1 has low catalytic activity that has the capacity to modulate sesB function. Dcp-1 and sesB may also be a part of a larger complex where Dcp-1 indirectly regulates the function and stability of sesB.

Given that sesB negatively regulates autophagic flux during mid-oogenesis, and that loss of Dcp-1 leads to increased sesB, we hypothesized that Dcp-1 acts upstream of sesB to regulate autophagy. Epistasis analyses revealed that *sesB^Org^;Dcp-1^Prev1^* double mutant flies contain degenerating mid-stage egg chambers similar to the *sesB^Org^* phenotype, placing Dcp-1 upstream of sesB in the regulation of autophagy. We propose a model where in response to starvation, Dcp-1 negatively regulates the levels of sesB in a nonproteolytic manner resulting in the reduction of ATP levels and an increase in autophagic flux. Further studies are required to determine the nature of the mechanism by which Dcp-1 modulates sesB levels and the identity of components acting both upstream and downstream in the pathway of Dcp-1-mediated autophagy.

Tissue-specific differences exist with respect to the requirement for Dcp-1 during starvation-induced autophagy. In the larval fat body, autophagy induction still occurred in response to starvation in *Dcp-1^Prev1^* flies although it is delayed relative to control flies. Is this differential requirement in regulating autophagy associated with the Dcp-1 cell death function that occurs in mid-stage egg chambers but not the fat body? Perhaps Dcp-1 functions as part of an early checkpoint decision, responding to environmental conditions, that helps determine the fate of mid-stage egg chambers to autophagy or apoptosis. It is possible that Dcp-1 acts as such a checkpoint regulator in additional tissues, but it remains unknown at this time.

While both *Drosophila* and mammalian pro- and cleaved caspases have been observed to localize to the mitochondria, little is known about their functions within this organelle. Several lines of evidence indicate that pro-caspases are not latent enzymes, highlighting that mitochondrially localized pro-Dcp-1 may have other important physiological functions in addition to the regulation of autophagy via sesB. In summary, our study shows that Dcp-1 has a novel, nonapoptotic role during mid-oogenesis where its mitochondrial localization is important for the maintenance of mitochondrial homeostasis under basal conditions and for the regulation of autophagic flux in nutrient-stress conditions.

## Notes

DeVorkinLGoNEHouYCMoradianAMorinGBGorskiSM The Drosophila effector caspase Dcp-1 regulates mitochondrial dynamics and autophagic flux via SesB J Cell Biol 2014 205 477 92 10.1083/jcb.201303144

